# FiCli, the Fish and Climate Change Database, informs climate adaptation and management for freshwater fishes

**DOI:** 10.1038/s41597-020-0465-z

**Published:** 2020-04-21

**Authors:** Trevor J. Krabbenhoft, Bonnie J. E. Myers, Jesse P. Wong, Cindy Chu, Ralph W. Tingley, Jeffrey A. Falke, Thomas J. Kwak, Craig P. Paukert, Abigail J. Lynch

**Affiliations:** 1 Department of Biological Sciences and the RENEW Institute, University at Buffalo, Buffalo, NY 14260 USA; 2 U.S. Geological Survey, National Climate Adaptation Science Center/North Carolina Cooperative Fish and Wildlife Research Unit, Department of Applied Ecology, North Carolina State University, Raleigh, NC 27695 USA; 3 Department of Environmental Science and Policy, George Mason University, Fairfax, VA 22030 USA; 4 Aquatic Resources and Monitoring Section, Ontario Ministry of Natural Resources and Forestry, Peterborough, ON K9L 0G2 Canada; 5 Missouri Cooperative Fish and Wildlife Research Unit, The School of Natural Resources, 302 Anheuser-Busch Natural Resources Building, University of Missouri, Columbia, MO 65211 USA; 6 U.S. Geological Survey, Alaska Cooperative Fish and Wildlife Research Unit, University of Alaska Fairbanks, P.O. Box 757020, Fairbanks, AK 99775 USA; 7 U.S. Geological Survey, North Carolina Cooperative Fish and Wildlife Research Unit, Department of Applied Ecology, North Carolina State University, Raleigh, NC 27695 USA; 8 U.S. Geological Survey, Missouri Cooperative Fish and Wildlife Research Unit, The School of Natural Resources, 302 Anheuser-Busch Natural Resources Building, University of Missouri, Columbia, MO 65211 USA; 9 U.S. Geological Survey, National Climate Adaptation Science Center, 12201 Sunrise Valley Drive, MS-516, Room 2A128A, Reston, VA 20192 USA

**Keywords:** Water resources, Ichthyology, Climate-change ecology

## Abstract

Inland fishes provide important ecosystem services to communities worldwide and are especially vulnerable to the impacts of climate change. Fish respond to climate change in diverse and nuanced ways, which creates challenges for practitioners of fish conservation, climate change adaptation, and management. Although climate change is known to affect fish globally, a comprehensive online, public database of how climate change has impacted inland fishes worldwide and adaptation or management practices that may address these impacts does not exist. We conducted an extensive, systematic primary literature review to identify peer-reviewed journal publications describing projected and documented examples of climate change impacts on inland fishes. From this standardized Fish and Climate Change database, FiCli (pronounced fick-lee), researchers and managers can query fish families, species, response types, or geographic locations to obtain summary information on inland fish responses to climate change and recommended management actions. The FiCli database is updatable and provides access to comprehensive published information to inform inland fish conservation and adaptation planning in a changing climate.

## Abstract (summary)

**Table:** 

Measurement(s)	Adaptation • response to climate change • freshwater fishes • management practices
Technology Type(s)	digital curation
Factor Type(s)	geographic location • date range of study
Sample Characteristic - Organism	fish
Sample Characteristic - Environment	climate system • lake • stream • wetland ecosystem • freshwater biome
Sample Characteristic - Location	Europe • Asia • Africa • North America • South America • Australia • Oceania

Machine-accessible metadata file describing the reported data: 10.6084/m9.figshare.12053385

## Background & Summary

Freshwater fishes have important subsistence, cultural, and economic value worldwide (Lynch *et al*.)^1^. However, freshwater ecosystems are among the most threatened on the planet with freshwater fishes showing the highest extinction rate among vertebrates in the 20th century (Burkhead 2012)^2^. The key anthropogenic pressures facing freshwater ecosystems are water extraction, habitat degradation, overexploitation, invasive species, and climate change (Reid *et al*.)^3^. These pressures occur at local, regional, and global scales and can have broad impacts on the life history, demography, and distribution of freshwater fishes (Costello 2015, Arthington *et al*.)^4,5^. In many cases freshwater fishes are limited in their ability to shift their distributions in response to climate change, increasing the vulnerability of many populations relative to marine fishes or terrestrial species. The value of inland freshwater fisheries has also been overshadowed by the magnitude of marine fisheries, but recent studies are revealing the value of these fisheries worldwide (Lynch *et al*.)^1^.

Systematic literature reviews in aquatic ecology provide information on large scale trends in ecosystem change, broader impacts of effects across diverse taxa and geographic location, and are especially important in management and policy-making (Haddaway *et al*.)^6^. While meta-analyses presented in these review papers are useful, extracting the underlying data from primary studies may be difficult because the database of original papers and information contained in the review paper are not readily accessible. Such reviews on the impacts of climate change on inland fishes have revealed increasing documentation of the effects of changes in temperature, precipitation, and other climate variables on fishes, but the effects are often interacting and complex. As a result, it can be difficult to capture all the dynamics and nuances in a single review paper (Comte *et al*., Lynch *et al*., Kovach *et al*., Myers *et al*.)^7–10^.

The creation of the Fish and Climate Change database (FiCli; pronounced fick-lee) draws from our personal attempts to systematically interpret information on climate change effects on inland fishes. We found little standardized information with which to evaluate responses across taxa, functional groups, and geographic regions. Therefore, we extracted and synthesized data from individual studies using a consistent format as a means to allow users to quantitatively assess broader relationships and make inferences, particularly for understudied species and regions. Potential adaptation or management actions have also been incorporated into the database when available. One of the major assets of FiCli is the built-in flexibility to add new studies. As these studies are integrated, our confidence in the responses, relationships, and metrics will increase and strengthen the ability of this resource to inform science-based management and adaptation planning.

**Fig. 1:**
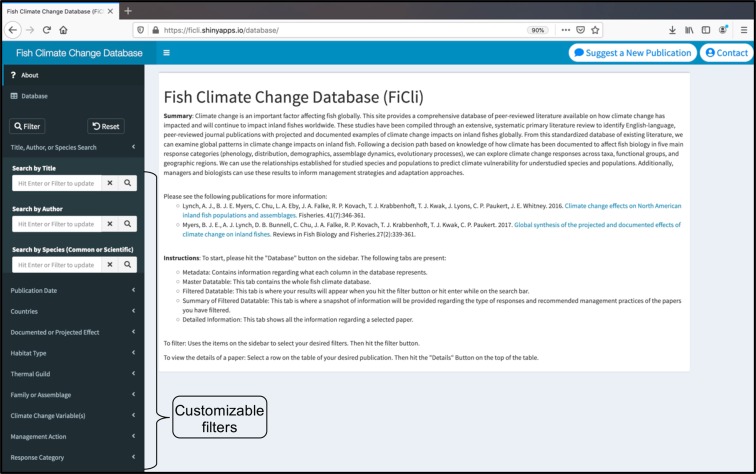
Graphical user interface for Fish and Climate Change (FiCli) database. Filters are described in the Data Records section.

**Fig. 2:**
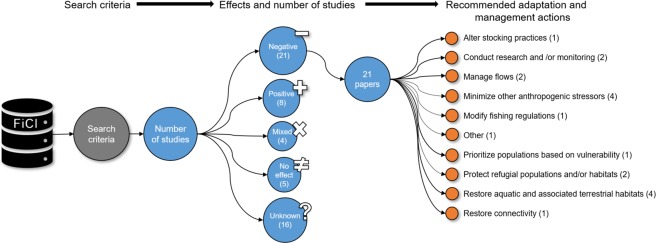
An example of graphical output available from a FiCli database query. Here the database was filtered for documented effects of climate change on coldwater salmonids and recommended adaptation actions. This graphical output is available to users following a specified search via the “Summary of Filtered Datatable” tab.

**Fig. 3:**
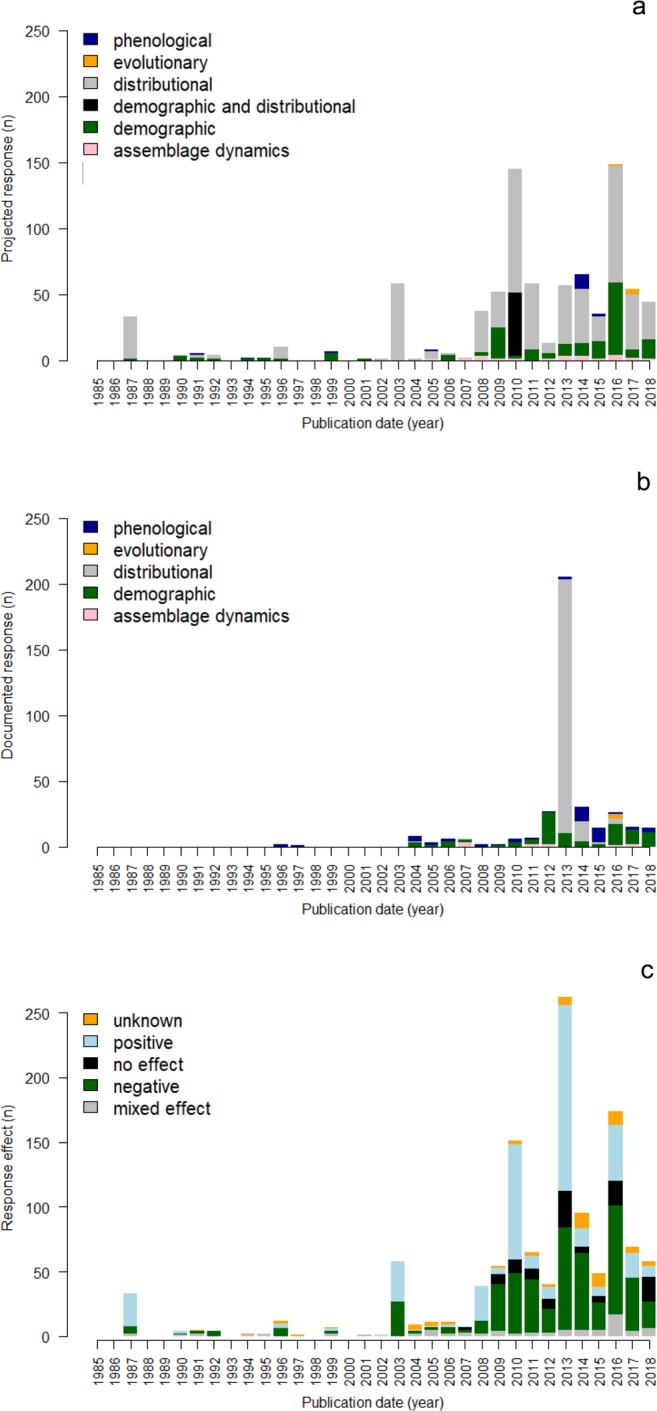
Published effects of climate change on inland fishes over time, split across response categories. (**a**) Projected responses, (**b**) Documented responses, (**c**) Responses illustrating no effect or a positive, negative, mixed, or unknown benefit of the response.

The goal for the FiCli database is to inform the development of conservation, management, and climate adaptation plans for inland fishes that integrate and prioritize scientific, logistical, financial, or regulatory actions when appropriate. The FiCli database was created using an established systematic literature review process with predefined inclusion criteria and multiple rounds of review and data extraction by fisheries experts (Myers *et al*.)^10^. With at least 15,000 freshwater fish species and 170 taxonomic families identified worldwide (Lévêque *et al*.)^11^, there remain significant geographic, taxonomic, and biological gaps in our understanding of climate change effects on freshwater fishes globally (Myers *et al*.)^10^. The FiCli database currently includes information for 53 freshwater fish families, 232 studies from over 47 countries, and 851 projected and 377 documented responses of individual species or assemblages to climate change. Each entry in the FiCli database includes the species studied, location, thermal guild classification, biotic metric studied, and direction of biotic metric response (Fig. 1). The database can be queried to assess the vulnerability of species to climate change given changes in their evolution, demography, distribution, assemblage dynamics, or phenology, and to identify adaptation and management actions that are recommended in the scientific literature to address those impacts (Figs. 2 and 3). Users can also summarize whether the documented or projected fish responses to climate change could have a positive, negative, mixed, or unknown benefit to the species, population, or assemblage (Fig. 3). This unique synthesis currently comprises projected and documented studies between 1985 and 2018 and can serve to focus species, population, assemblage, or ecosystem research priorities because different filters can be applied to identify knowledge gaps. Informed predictions about climate change effects for under- or unrepresented species can then be generated using projections and observations among conspecifics or surrogate species or ecosystems. The FiCli is a living database that will be updated by its curators as new studies are published, and users also have the opportunity to inform curators of peer-reviewed publications that should be considered for addition to the database.

## Methods

### Database, R-Shiny and Decision Path Tool

The FiCli database was generated from a review of primary scientific literature from 1985 to 2018. The year 1985 was a conservative choice for representing the earliest publications of effects of climate change on fishes, based on initial searches. The database initially included primary journal articles on climate change effects for North American inland fishes (Lynch *et al*.)^8^ and was then expanded to include global coverage for publications that explicitly assessed the projected and documented effects of climate change on inland fishes (Myers *et al*.)^10^. These papers were partitioned into studies documenting effects of climate change on fishes (“documented effects,” generally based on long-term datasets) or through projections of future effects (“projected effects,” often by applying modeling approaches). Some studies included both documented and projected effects.

We present a brief description of the criteria used for inclusion of papers in the database based on previous literature reviews (Lynch *et al*., Myers *et al*.)^8,10^. Our literature review included multiple rounds of review by team members to ensure appropriateness for inclusion in the dataset. We then re-reviewed all papers in the database to identify suggested management recommendations and a binary response category to illustrate whether the species or population exhibited, or were projected to exhibit, a positive, negative, mixed, unknown, or no effect of climate change. For example, increases in growth and distribution were considered to positively benefit the species, while earlier or later spawning time was considered to be an unknown effect. This binary response category is a coarse indicator of direction of response to climate change and was determined based on the interpretation of results presented in the reviewed paper and our best judgement. For ease of interpretability, the management recommendations were binned into eleven overarching categories (Table 1).

**Table 1:** List of the 11 adaptation and management recommendation categories in FiCli with associated examples from the literature.

Binned Adaptation and Management Recommendations	Example from Literature
Prioritize populations based on vulnerability	Promote thermal-tolerance diversity when prioritizing metapopulations for conservation (Anderson *et al*.)^15^
Manage flows	Work with dam managers to optimize reservoir release schedules (Segurado *et al*.)^16^
Restore connectivity	Maintain connectivity to inlet streams to promote cooling (Griffiths and Schindler 2012)^17^
Protect refugial habitats	Protect deep pools, headwater streams, and springs (Ries and Perry 1995)^18^
Restore aquatic and associated terrestrial habitats	Maximize riparian vegetation shading to reduce solar input and increase allochthonous prey input (McCarthy *et al*.)^19^
Minimize other anthropogenic stressors	Conduct fuel management to reduce fire size (Falke *et al*.)^20^
Alter stocking practices	Manipulate temperature in hatcheries to avoid shifts in sex ratio due to environmental sex determination (Wedekind and Küng 2010)^21^
Modify fishing regulations	Modify fishing season dates to match shifts in migration timing (Valiente *et al*.)^22^
Conduct research and/or monitoring	Develop phenotype-environment associations for species to predict adaptive potential (Michel *et al*.)^23^
Education and outreach	Development of prevention strategies to deter new non-native species introductions (Milardi *et al*.)^24^
Other	Use factors beyond thermal guild to predict effects of climate change on fish growth (Mills *et al*.)^25^

In order to allow public access to the database, the FiCli database is available as a R-Shiny application supported by the U.S. Geological Survey (USGS), National Climate Adaptation Science Center (available here: https://ficli.shinyapps.io/database/) and does not require installation of the R program. All data and the R-Shiny application code are made available by USGS (10.5066/P9VKJ23R). The online FiCli database is dynamic, and new papers will continue to be added on a rolling basis as they are published. A web upload form on the R-shiny site allows user-submitted database entries that will be reviewed by the FiCli team and added as appropriate. Future plans include: (1) expansion and re-release of the FiCli database to include studies with a primarily marine focus, and (2) inclusion of papers that use experimental approaches to describe fish response to changes in temperature and flow in the context of climate change; such studies were initially excluded from ours and previous literature reviews (Lynch *et al*., Myers *et al*.)^8,10^.

## Data Records

The FiCli database includes summarized data on study systems, species, climate change responses, and adaptation and/or management actions into standardized categories that can inform species vulnerability assessments and adaptation planning (Online-only Table 1). Information was extracted from each peer-reviewed publication that the author team identified for the systematic literature review. A series of filters have been embedded into the application for customizable searches (Online-only Table 1). A graphic description of the results can be generated within the R-shiny application (Fig. 2), and detailed summaries can be exported as Bibtex or Ris files. The original raw data tables used to create the FiCli database are also available on Figshare (Krabbenhoft *et al*.)^12^.

## Technical Validation

The literature compilation and summary presented in the FiCli database have been reviewed and validated by multiple approaches. Each publication included in the database has been reviewed for content at two levels: (1) a cursory inspection for applicability and (2) a detailed review to extract relevant information entered into summary categories (i.e., database columns). The information included in each reference entry (i.e., database rows) was then reviewed by another author of the database.

Many of the references included in the database formed the basis of two recent primary literature synthesis publications by this group of authors. The first publication (Lynch *et al*.)^8^ synthesized climate trends that influence North American inland fish populations and assemblages and included case studies illustrating the discerned trends. The second publication (Myers *et al*.)^10^ was a global synthesis of documented and projected climate change effects on inland fishes. Thus, the literature references provided in the database have undergone review for accuracy and content by multiple authors of this publication, internal reviewers of these three publications, and journal referees of each publication. The authors who have collaborated on this and the previous two publications represent a diverse background of expertise (multiple fields in conservation and ecology), professional affiliation (natural resource agency or academia), and geographic location (throughout the United States of America and Canada).

The USGS, an affiliation of multiple authors, requires internal review of science products, including manuscripts for publication and data incorporated into publications. The database contents were reviewed and made publicly available through a Data Management Plan required of all USGS science products, and the manuscript was reviewed internally in adherence to the Fundamental Science Practices process. The USGS review process is intended to ensure and enhance the quality, accuracy, and availability of all science products released to the public and scientific community.

The references included in the database were derived through a systematic literature review, and information included in the database has been intensively reviewed. Though the literature included was considered thorough, it is unlikely to be exhaustive, especially as new science is published. Semi-annual updates to the database are planned. The most recent update date will be provided in the metadata. Further, we summarize key information extracted from individual publications on the documented or projected impacts of climate change on inland fish globally, but we have not reviewed each publication for accuracy and rely on the journal peer-review process to ensure scientific quality, accuracy, and integrity.

In conclusion, climate change will continue to impact inland fish populations and assemblages, productivity, and overall biodiversity (Diaz *et al*., Till *et al*.)^13,14^. The full extent of the consequences of climate change on inland waters is yet to be known, but inland fish and fisheries are changing with implications for human use of these natural resources. The FiCli database will make the most up-to-date peer-reviewed information of climate change impacts on inland fish easily accessible and will serve to provide information to managers and policy makers on actions to enhance climate change adaptation in inland waters.

## Data Availability

Code is available for download at 10.5066/P9VKJ23R. There are no restrictions to the access or use of this code. Code was implemented in R (version 3.6.1; https://r-project.org) using the package *shiny* (version 1.3.2).

## Online-only Table

**Online-only Table 1:** Data records stored in the Fish and Climate Change (FiCli) database; a global synthesis of the documented and projected impacts of climate change on freshwater fish species.

Data field	Definition
Author*	Last name of first author or first and second authors.
Title*	Keyword search through paper titles.
Documented or projected*	Identifies whether climate change response was observed (documented) or projected based on climate change simulations.
Publication date*	Year study was published.
Date range of study	Beginning and ending years of documented studies or time periods for projected studies.
Study area	Geographic location of study. This can be a specific lake or river name, or a region if multiple water bodies.
Habitat type*	Stream, lake (including reservoirs), or wetland.
Lentic or lotic	Still or flowing freshwater habitat.
Climate change variable*	Physical climate variable (temperature, precipitation, flow, ice cover).
Focal species or assemblage*	Study species or fish assemblage.
Additional species	Species that were studied in the publication but were not the main or focal species. The additional species field includes data when the response was assemblage dynamics.
Response category*	Ecological or biological process reported.
Thermal guild*	Cold-, cool-, or warm-water species.
Family or fish assemblage*	The taxonomic family of the fish studied. If the study was focused on an assemblage, then family is classified as “fish assemblage”.
Continent/Region	The continental location or geographic region where the study was conducted.
Country*	The country or countries where the study was conducted.
Response	A generalized description of the change in the fish response variable reported in each individual study (increase, decrease, shift, no change).
Binary response*	Categorization of the change in the response to illustrate whether the change is positive, negative, mixed, no effect, or unknown.
Management recommendations	Ten categories of potential management actions that could be taken (alter stocking practices, conduct research and/or monitoring, education and outreach, manage flows, minimize other anthropogenic stressors, modify fishing regulations, prioritize populations based on vulnerability, protect refugial populations and/or habitats, restore aquatic and associated terrestrial habitats, restore connectivity).

*Highlights the filters that can be used in FiCli to generate customized summaries of the effects of climate change on freshwater fishes.
